# Longitudinal association between depressive symptoms and cognitive function: the neurological mechanism of psychological and physical disturbances on memory

**DOI:** 10.1017/S0033291724001612

**Published:** 2024-10

**Authors:** Xiangwei Dai, Sihan Liu, Xin Li, Kewei Chen, Shudan Gao, Jun Wang, Dag Aarsland, Zhuo Rachel Han, Zhanjun Zhang

**Affiliations:** 1 State Key Laboratory of Cognitive Neuroscience and Learning & IDG/McGovern Institute for Brain Research, Beijing Normal University, Beijing, China; 2 Institute of Basic Research in Clinical Medicine, China Academy of Chinese Medical Sciences, Beijing, China; 3 BABRI Centre, Beijing Normal University, Beijing, China; 4 Faculty of Psychology, Beijing Key Laboratory of Applied Experimental Psychology, National Demonstration Center for Experimental Psychology Education, Beijing Normal University, Beijing, China; 5 Banner Alzheimer's Institute, Phoenix, Arizona, USA; 6 School of Psychology, Shandong Normal University, Jinan, Shandong, China; 7 Department of Old Age Psychiatry, Institute of Psychiatry, Psychology and Neuroscience, King's College London, London, UK; 8 Center for Age-Related Diseases, Stavanger University Hospital, Stavanger, Norway

**Keywords:** chronic vascular diseases, episodic memory, late-life depressive symptoms, MRI, thalamus, white matter integrity

## Abstract

**Background:**

The neural correlates underlying late-life depressive symptoms and cognitive deterioration are largely unclear, and little is known about the role of chronic physical conditions in such association. This research explores both concurrent and longitudinal associations between late-life depressive symptoms and cognitive functions, with examining the neural substrate and chronic vascular diseases (CVDs) in these associations.

**Methods:**

A total of 4109 participants (mean age = 65.4, 63.0% females) were evaluated for cognitive functions through various neuropsychological assessments. Depressive symptoms were assessed by the Geriatric Depression Scale and CVDs were self-reported. T1-weighted magnetic resonance imaging (MRI), diffusion tensor imaging, and functional MRI (fMRI) data were acquired in a subsample (*n* = 791).

**Results:**

Cognitively, higher depressive symptoms were correlated with poor performance across all cognitive domains, with the strongest association with episodic memory (*r* = ‒0.138, *p* < 0.001). Regarding brain structure, depressive symptoms were negatively correlated with thalamic volume and white matter integrity. Further, white matter integrity was found to mediate the longitudinal association between depressive symptoms and episodic memory (*indirect effect* = −0.017, 95% CI −0.045 to −0.002) and this mediation was only significant for those with severe CVDs (*β* = −0.177, *p* = 0.008).

**Conclusions:**

This study is one of the first to provide neural evidence elucidating the longitudinal associations between late-life depressive symptoms and cognitive dysfunction. Additionally, the severity of CVDs strengthened these associations, which enlightens the potential of managing CVDs as an intervention target for preventing depressive symptoms-related cognitive decline.

## Introduction

Late-life depressive symptoms refer to a subthreshold condition in which individuals experience symptoms similar to major depressive disorder without meeting the diagnostic criteria (Biella et al., 2019). As a commonly reported psychiatric condition in older populations, late-life depressive symptoms can cause distress and impact daily functioning. Prior studies have suggested that late-life depressive symptoms are closely associated with cognitive deterioration and the incidence of neurodegenerative diseases such as Alzheimer's disease (AD) (Butters et al., 2008; Kaup et al., 2016). Additionally, late-life depressive symptoms are frequently comorbid with various chronic diseases, especially chronic vascular diseases (CVDs) (Alexopoulos, 2005). Growing research has indicated that comorbidity might play an important role in the deterioration of cognitive function in older adults (Kim & Han, 2021; Vu & Aizenstein, 2013). However, it is still unclear how the comorbidity of psychological and physical disturbances deteriorates cognitive performance on the neurological level.

Recent neuroimaging studies have shown that individuals with depression experience significant dysfunction in brain networks (Gong & He, 2015). Aberrant white matter (WM) alterations such as disrupted WM integrity are regarded as a primary neurobiological substrate of depression during aging (Reppermund et al., 2014; Sexton et al., 2012), which may provide a structural basis for the network dysfunctions through disconnecting neural circuits (Smagula & Aizenstein, 2016). Moreover, WM microstructure impairments are also reported to underlie depression-derived cognitive dysfunction (Lee et al., 2022; Xu et al., 2020). Similarly, patients with CVDs, such as coronary artery disease and hypertension, also show microstructural WM abnormalities (Wardlaw, Smith, & Dichgans, 2019). These CVDs-related WM microstructure impairments may lead to disconnections in cognitive networks, causing further exacerbation of cognitive impairments (Kim & Han, 2021; Veldsman et al., 2020). Therefore, CVDs might worsen the neural correlates linking late-life depressive symptoms and cognitive function by damaging the WM structure. However, previous longitudinal studies on the influence of late-life depressive symptoms on cognition were mostly carried out without considering the interaction between depressive symptoms and CVDs. In addition, the neural correlates underlying this interaction have been largely unclear. Thus, the neural correlates of psychological and physical disturbances on cognitive function warrant further examination with multimodal MRI data.

Our study is one of the first attempts to delineate the neurological pathways between late-life depressive symptoms and CVDs on cognitive function. Using multimodal imaging techniques, we first explore which neural pathways of depressive symptoms impact cognitive function by examining the symptoms-derived brain structural/functional alterations. Then, we examine the moderating role of CVDs on the above-mentioned pathways using both concurrent and longitudinal data, to identify whether the influences of late-life depressive symptoms on cognitive functions differ among individuals with various severity of CVDs. We hypothesize that severe CVDs would strengthen the association between late-life depressive symptoms and WM structure, exaggerating longitudinal cognitive decline for those with such chronic conditions. This work could advance our understanding of the comorbidity of psychological and physical disturbances on cognitive dysfunction during aging and provide a novel perspective to preventing cognitive decline in the older population.

## Methods

### Participants

Participants were recruited from the Beijing Aging Brain Rejuvenation Initiative (BABRI), a community-based cohort study aiming to examine the brain and cognitive changes during aging (Yang et al., 2021). Inclusion criteria for BABRI participants were: aged 50 years old or older, no clinical diagnoses of psychiatric disorders (e.g. major depression disorder, bipolar disorder), no history of neurological disorders, no impairments of visual and hearing function, and able to complete various neuropsychological tests.

The BABRI study adopts an accelerated longitudinal design. Since its initiation in 2008, this study conducts follow-ups every 2–3 years, while recruiting new participants concurrently. For the present study, we used data from baseline and first follow-up for all participants in the BABRI cohort. This encompassed a total of 4109 participants, with a mean age of 65.4 years (standard deviation, s.d. = 7.5), 63.0% females, and an average education length of 11.1 years (s.d. = 3.3) (online Supplementary Table S1). For a comprehensive exploration of neuroimaging characteristics associated with late-life depressive symptoms and the neural correlates underlying depressive symptoms-related cognitive changes, we focused on the MRI sub-cohort (*n* = 791). Within this sub-cohort, 327 participants were available for neuropsychological follow-up at a mean interval of 1.89 years, allowing longitudinal analysis of cognitive data. There were no differences in main variables including demographic information, episodic memory performance, and neuroimaging indicators between the MRI and longitudinal samples, except for the incidence of hyperlipidemia and the total number of diseases (online Supplementary Table S2).

This study was approved by the Ethics Committee and Institutional Review Board of Beijing Normal University's Imaging Centre for Brain Research (ICBIR_A_0041_002_02), and all participants completed written informed consent.

### Questionnaires and neuropsychological assessment

All participants completed a personal information questionnaire and a series of neuropsychological tests (Yang et al., 2021). The basic personal information included demographic (i.e. age, sex, educational level) and disease (i.e. diabetes, hypertension, hyperlipidemia, cerebrovascular disease, heart disease) information. Trained researchers rated yes/no to each question based on participants’ self-reported medical history and prescriptions. Additionally, participants reported their depressive symptoms using the 30-item Geriatric Depression Scale (GDS) (Yesavage et al., 1982). Each item was a yes/no question and a score of 0 or 1 could be assigned to each answer. The GDS score ranged from 0 to 30, with higher scores indicative of more severe depressive symptoms. The participants were then divided into three groups based on their GDS scores. Individuals with GDS scores of 0–10 were in a normal state, 11–20 indicated mild-to-moderate depressive symptoms, and > 20 was ssuggestive of severe depressive symptoms. Also, a set of neuropsychological assessments were applied to evaluate cognitive function. The general cognitive function was tested using the Chinese version of the Mini-Mental State Examination (MMSE) (Zhang et al., 1990); memory was tested using the Auditory Verbal Learning Test (AVLT) (Guo, Lu, & Hong, 2001), the Digit Span Test (a sub-test of the Wechsler Adults Intelligence Scale-Chinese revision); and the Rey-Osterrieth Complex Figure Test (ROCF) (Rey, 1941); executive function was tested using the Stroop Color-Word Test (SCWT) (Guo et al., 2005) and the Trail Making Test (TMT) (Reitan, 1958); spatial processing was tested using the Clock Drawing Test (CDT) (Rouleau, Salmon, Butters, Kennedy, & McGuire, 1992) and the ROCF_Copy test (Rey, 1941); attention was tested using the Symbol Digit Modification Test (SDMT) (Sheridan et al., 2006) and the TMT_A test (Reitan, 1958); language was tested using the Boston Naming Test (BNT) (Guo, Hong, Shi, Sun, & Lv, 2006) and the Verbal Fluency Test (VFT) (Mok, Lam, & Chiu, 2004).

### MRI image acquisition

MRI data were acquired by a SIEMENS TRIO 3 T scanner in the Imaging Center for Brain Research, Beijing Normal University. Participants were in a supine position with their heads snugly fixed by straps and foam pads to minimize head movement. The T1-weighted structural images were acquired using 3D magnetization-prepared rapid gradient echo sequences: (192 sagittal slices, repetation time (TR) = 2300 ms, echo time (TE) = 2.32 ms, slice thickness = 0.90 mm, flip angle = 8°, field of view  (FOV) = 240 mm × 240 mm). The DTI images were acquired using a single-shot echoplanar imaging sequence: (coverage of the whole brain, 2 mm slice thickness with no interslice gap, 75 axial slices, repetition time = 8000 ms, echo time = 60 ms, flip angle = 90°, 30 diffusion directions with *b* = 1000 s/mm^2^, and an additional image without diffusion weighting, acquisition matrix = 128 × 128). Resting-state fMRI data were collected using a gradient echo EPI sequence: (TE = 30 ms, TR = 2000 ms, flip angle = 90°, 33 slices, slice thickness = 4 mm, in-plane matrix = 64 × 64, FOV = 256 × 256 mm). During the single-run resting acquisition, the subjects were instructed to remain awake, relax with their eyes closed, and remain as motionless as possible. The resting acquisition lasted for 8 min, and 240 image volumes were obtained.

### Data preprocessing

MATLAB 2012b (MathWorks, MA, USA) and SPM12 software package (https://www.fil.ion.ucl.ac.uk/spm) with default settings were used to preprocess and spatially normalize the structural images, as well as to segment the T1 images to gray matter (GM). The modulated GM images were smoothed with a Gaussian kernel of 8 mm full-width half-maximum (FWHM). We utilized the mean GM map (threshold = 0.2) of all the participants to generate a group brain mask and used it for subsequent analysis.

The FMRIB software library (FSL, https://fsl.fmrib.ox.ac.uk/fsl/fslwiki/) was utilized to preprocess the DTI data. This preprocessing includes (1) converting DICOM files to the NIfTI format, (2) estimating the brain mask, (3) removing non-brain space, (4) correcting the eddy current and head motion, (5) adjusting diffusion gradient directions, and (6) calculating the voxel-wise DTI metrics. We conducted a rigorous visual inspection throughout the preprocessing to ensure data quality and registrations. Additionally, an atlas-based approach explored diffusion changes in specific tracts. We used the ICBM-DTI-81 WM labels atlas to segment the entire WM into 48 regions of interest (ROIs). The atlas-based fractional anisotropy (FA) and mean diffusivity (MD) were further calculated by averaging the values within each region of the WM atlas.

The fMRI data were preprocessed using SPM12 and DPABI (http://rfmri.org/DPABI) (Yan, Wang, Zuo, & Zang, 2016). The processing included removing the first 10 volumes, slice timing, within-subject interscan realignment to correct for possible movement, spatial normalization, resampling to 3 × 3 × 3 mm, and smoothing with an 8 mm FWHM Gaussian kernel. Independent component analysis (ICA) was performed using the group ICA of fMRI toolbox (GIFT version 2.0; http://mialab.mrn.org/software/gift/). There are three main stages of the GIFT procedure: (1) data reduction by principal component analysis for each subject for data reduction, (2) application of the ICA algorithm, and (3) back-reconstruction for each individual subject. Thirty components were estimated in this study. By visual inspection and correlating each component with the given templates, the best-fit component was selected which represented each of the default mode network (DMN), the left frontal-partial network (LFP), and the right frontal-partial network (RFP), respectively. The spatial maps of each of these selected components were then compared between the three groups.

### Statistical analysis

Data analysis was conducted with *SPSS* 25.0, *Mplus* 8.3, and the *cocor* package (Diedenhofen & Musch, 2015). We first identified which cognitive domains and types of diseases were closely related to depressive symptoms. After controlling for age, sex, and educational level, a series of analyses of variance (ANOVAs) and partial correlation analyses were applied to first compare cognitive performance between groups with different levels of depressive symptoms and then explore the strength between depressive symptoms and cognitive performance within each cognitive domain. Additionally, Fisher *z*-transformation was applied to all the correlation coefficients which were followed by a *Z*-test to further determine the most vulnerable cognitive domain to depressive symptoms. In addition, the binary logistic regression analysis model was built to determine whether exposure to diseases is a risk factor for depressive symptoms with age, sex, and years of education controlled as covariates.

Using SPM, voxel-wise comparisons of gray matter volume and functional connectivity (FC) within DMN, LFP, and RFP were performed between the three groups of different levels of depressive symptoms. The threshold for the resulting statistical parametric maps was established at a voxel-wise at *p* < 0.001 (uncorrected), and then GRF-corrected was used for multiple comparisons. The DTI metrics of the atlas-based tract ROIs were analyzed using analysis of covariance (ANCOVA) where the age, sex, and years of education were controlled. *Post-hoc* pairwise *t* tests with Bonferroni correction were conducted when the ANCOVA results were statistically significant after multiple comparisons.

Moreover, structural equation modeling (SEM) using maximum likelihood estimation was tested to explore the associations among depressive symptoms, CVDs, brain alterations, and both concurrent and longitudinal cognitive performance, with age, sex, educational level, and collection interval serving as covariates. Before the model testing, the model structure was defined as follows: depressive symptoms were an observed variable using the total score of GDS; CVDs were an observed variable by summing up the disease occurrences; brain alterations and cognitive performance were latent variables loaded by the specific indicators most related to depressive symptoms. Online Supplementary Fig. S1 describes the flow chart of the SEM, including three common steps suggested by previous studies (Liu, You, Ying, Li, & Shi, 2020): (a) a measurement model with latent variables; (b) a mediation model of brain alterations between late-life depressive symptoms and cognitive performance, bootstrapped 1000 times; (c) a moderated mediation model to test the moderating effect of disease on the paths between late-life depressive symptoms and brain alterations. Model fit indices included a comparative fit index (CFI > 0.90), a Tucker–Lewis index (TLI > 0.90), and a standardized root mean square residual (SRMR < 0.06).

## Results

### Demographic and clinical characteristics of the cohort

A total of 4109 participants were enrolled in this study. The mean GDS score was 7.83 (s.d. = 6.1), with 20.9% of participants showing mild-to-moderate and 5.2% showing severe depressive symptoms. Online Supplementary Table S1 displays the distribution and prevalence of depressive symptoms and various diseases. Briefly, the prevalence of both mild-to-moderate and severe symptoms was higher in females than in males and decreased along with age. The prevalence of depressive symptoms ranges from 18.2% to 28.9%, and older adults with high levels of education exhibit a lower incidence of depressive symptoms. For the comorbidities, hypertension is the most common disease among older adults. In addition, the prevalence of all chronic diseases was relatively higher in the oldest group than in the other two younger groups, with the exception of hyperlipidemia.

### Neuropsychological characterizations of older adults with different severity of depressive symptoms

Table 1 displays group differences and associations between depressive symptoms and cognitive performance. In general, cognitive performance measures in all six domains, except spatial processing, showed significant differences among the normal, mild-to-moderate depressive symptoms, and severe depressive symptoms groups, even after adjusting for age, sex, and education. Subsequent analyses revealed that both the ‘severe depressive symptoms’ group and the ‘mild-to-moderate’ group scored significantly lower than the ‘normal’ group in most tests. Regarding correlation comparisons, we found that episodic memory exhibited the most pronounced influence from depressive symptoms among all the six cognitive domains measured (online Supplementary Table S3). Therefore, episodic memory was selected as the main focus in later analyses.

**Table 1.** Cognitive characteristics of the 4109 participants according to depressive symptoms status

	NC	Mild-to-moderate LDS	Severe LDS	*F*/χ^2^	*p*	Correlation with LDS	
	(*n* = 3038)	(*n* = 858)	(*n* = 213)			*r*	*p*
General cognition						/	/
MMSE	27.481 ± 2.36	27.114 ± 2.249	26.829 ± 2.724	13.198	<0.001^ab^	−0.113	<0.001
Episodic memory						−0.138	<0.001
AVLT_Delay	5.194 ± 2.583	4.739 ± 2.449	4.450 ± 2.502	22.665	<0.001^ab^	−0.119	<0.001
AVLT_Total	28.264 ± 9.222	26.115 ± 8.673	25.550 ± 8.560	31.178	<0.001^ab^	−0.133	<0.001
ROCF_Delay	12.787 ± 6.740	11.850 ± 6.395	10.868 ± 6.447	10.351	<0.001^ab^	−0.084	<0.001
Working memory						−0.050	0.003
DST	12.169 ± 2.299	11.768 ± 2.167	11.811 ± 2.073	6.514	0.001^a^	−0.051	0.002
DST-Backward	4.484 ± 1.337	4.251 ± 1.239	4.264 ± 1.214	7.090	0.001^a^	−0.042	0.012
Spatial processing						−0.046	0.006
ROCF_copy	33.280 ± 4.569	32.793 ± 4.728	33.179 ± 3.525	1.751	0.174	−0.042	0.012
CDT	23.830 ± 5.021	23.399 ± 4.873	23.269 ± 5.097	0.836	0.433	−0.029	0.078
Language						−0.073	<0.001
VFT	45.077 ± 9.051	43.401 ± 8.824	42.392 ± 9.503	18.514	<0.001^ab^	−0.100	<0.001
BNT	23.536 ± 3.756	23.126 ± 3.571	23.152 ± 3.640	0.946	0.388	−0.015	0.360
Processing speed						−0.102	<0.001
SDMT	34.032 ± 11.358	31.977 ± 10.395	31.172 ± 10.314	19.039	<0.001^ab^	−0.103	<0.001
TMT_A	60.129 ± 28.622	62.725 ± 27.903	65.707 ± 27.688	5.748	0.003^b^	0.066	<0.001
Executive function						−0.098	<0.001
SCWT-C-Time	78.200 ± 24.615	81.123 ± 28.930	84.627 ± 30.164	16.572	<0.001^ab^	0.096	<0.001
TMT-B	172.177 ± 71.587	178.925 ± 70.431	183.567 ± 75.872	4.682	0.009	0.058	<0.001

Corrected for age, sex, years of education. MMSE, Mini-Mental State Examination; AVLT, Auditory Verbal Learning Test; ROCF, Rey-Osterrieth Complex Figure Test; SCWT, Stroop Color Word Test; TMT, Trail Making Test; CDT, Clock Drawing Test; SDMT, Symbol Digit Modification Test; BNT, Boston Naming Test; VFT, Verbal Fluency Test; DST, Digital Span Test; LDS, late-life depressive symptoms; NC, 0 ⩽ GDS ⩽ 10; mild-to-moderate LDS, 10 < GDS ⩽ 20; severe LDS, 20 < GDS ⩽ 30.

^a^*Post-hoc* paired comparisons showed significant group differences between NC and mild-to-moderate LDS.

^b^*Post-hoc* paired comparisons showed significant group differences between NC and severe LDS.

^c^*Post-hoc* paired comparisons showed significant group differences between mild-to-moderate LDS and severe LDS.

### Vascular-related risk factors of late-life depressive symptoms

The binary logistic regression analysis was conducted to identify which type of disease was most related to depressive symptoms. The results showed that except for diabetes (a metabolic disease), all other CVDs observed in this study (hypertension, hyperlipidemia, cerebrovascular disease, and heart disease) predicted higher severity levels of depressive symptoms (online Supplementary Fig. S2). Consequently, these four diseases were summed up as the indicator of CVDs.

### Brain structural and functional characterizations of older adults with different severity of depressive symptoms

**Figure 1.**
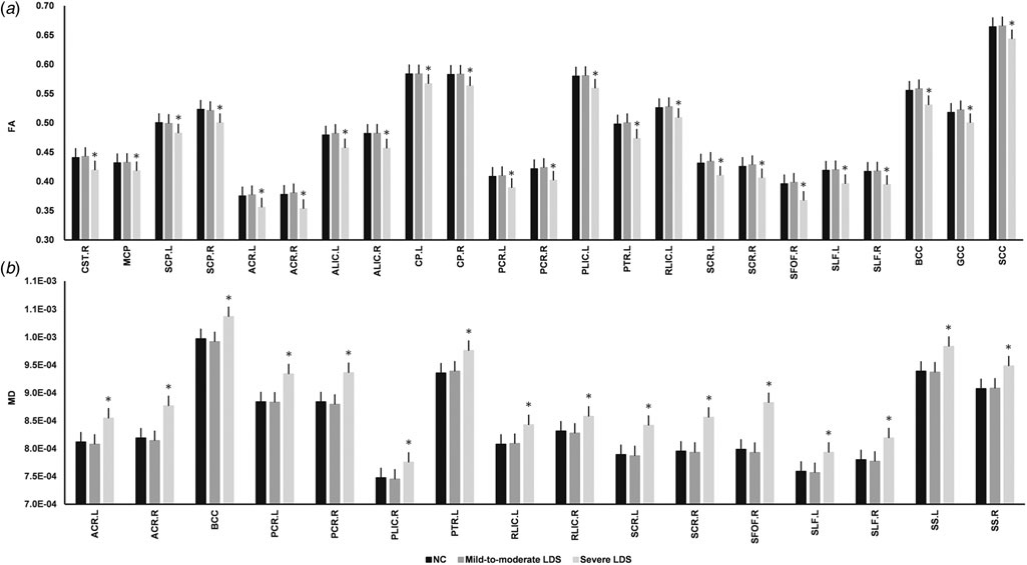
White matter integrity differences among the three depressive symptoms groups. (a) The fractional anisotropy (FA) and group differences of each atlas-based tract ROI in the three groups. (b) The mean diffusivity (MD) and group differences of each atlas-based tract ROIs in the three groups. Asterisk indicates a significant difference between the severe LDS group and the other two groups after Bonferroni correction. CP, cerebral peduncle; PCR, posterior corona radiata; PLIC, posterior limb of internal capsule; RLIC, retrolenticular part of internal capsule; ALIC, anterior limb of internal capsule; ACR, anterior corona radiata; PTR, posterior thalamic radiation; SFOF, superior fronto-occipital fasciculus; SCR, superior corona radiata; SLF, superior longitudinal fasciculus; SS, sagittal stratum; SCP, superior cerebellar peduncle; BCC, body of corpus callosum; GCC, genu of corpus callosum; SCC, splenium of corpus callosum; MCP, middle cerebellar peduncle; CST, corticospinal tract; LDS, late-life depressive symptoms; NC, 0 ⩽ GDS ⩽ 10; mild-to-moderate LDS, 10 < GDS ⩽ 20; severe LDS, 20 < GDS ⩽ 30.

**Figure 2.**
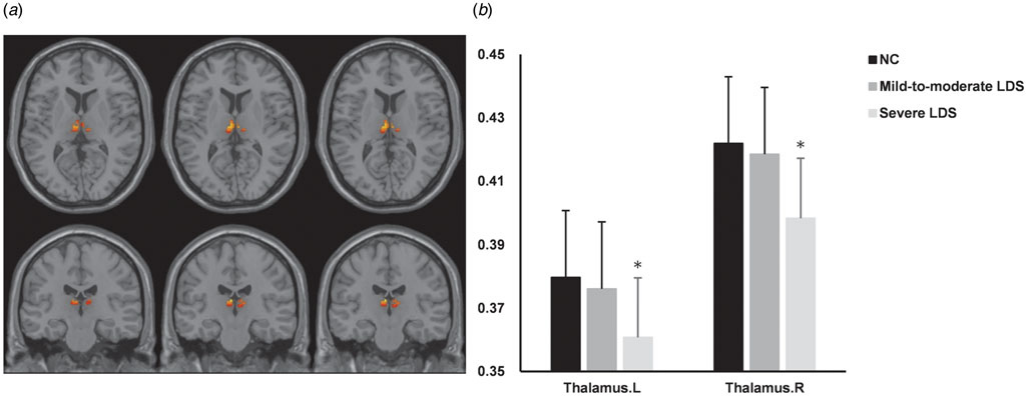
Gray matter volume differences among the three depressive symptoms groups. (a) Correlation between depressive symptoms and gray matter volume. (b) Thalamic volume differences between three groups of older adults. Corrected for age, sex, and years of education. Regions of reduction of gray matter correlated with late-life depressive symptoms increase are displayed in red for voxel-wise threshold of *p* < 0.001. Asterisk indicates a significant difference between the severe LDS group and the other two groups. LDS, late-life depressive symptoms; NC, 0 ⩽ GDS ⩽ 10; mild-to-moderate LDS, 10 < GDS ⩽ 20; severe LDS, 20 < GDS ⩽ 30.

Differences in DTI metrics of the atlas-based tract ROIs among the three groups were analyzed and are shown in Fig. 1 and online Supplementary Table S4. Older adults in the severe depressive group showed significantly decreased FA and increased MD in most ROIs. These tracts were mainly projection fibers connecting thalamic and cortical regions. In addition, Pearson analysis reported that the bilateral anterior limb of internal capsule and left posterior thalamic radiation were the most influenced tracts connecting to the thalamus (online Supplementary Table S5). As for the GM, the ANOVA analysis revealed significant bilateral thalamic atrophy in older adults with severe depressive symptoms, while no significant GM changes were found in older adults showing mild-to-moderate depressive symptoms (Fig. 2 and online Supplementary Table S6).

Regarding the resting-state FC analysis, we found no significant associations between the degree of depressive symptoms and FC levels in any of the selected components representing the DMN, LFP, or RFP networks. Besides, there were no significant group differences in network FC found between the three groups (online Supplementary Fig. S3).

### The moderating role of CVDs on the associations between depressive symptoms and concurrent and longitudinal episodic memory

Before the model testing, we explored the correlations between the severity of CVDs and depressive symptoms among the MRI and the longitudinal cognitive samples. At baseline, the severity of CVDs was significantly correlated with depressive symptoms (*n* = 791, *r* = 0.153, *p* = <0.001) in the MRI sub-cohort and showed a marginal correlation with depressive symptoms in the longitudinal cognitive sample (*n* = 327, *r* = 0.096, *p* = 0.084).

**Figure 3.**
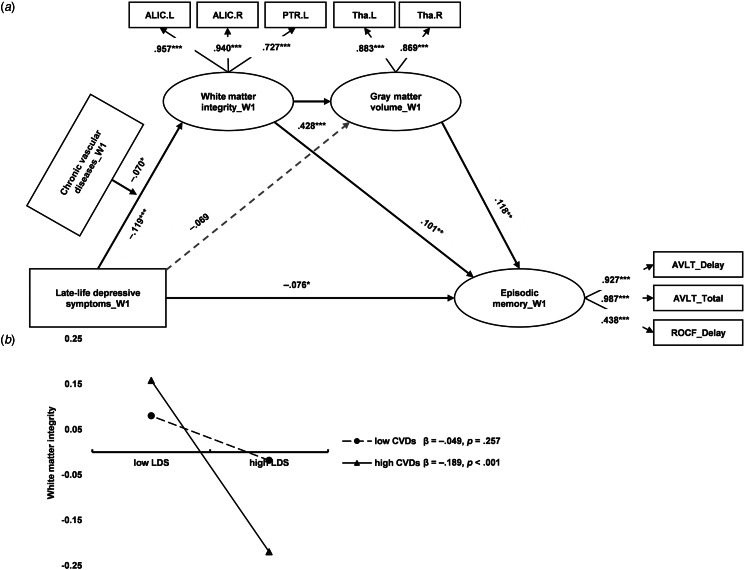
Chronic vascular disease (CVDs) moderates the neural correlates of late-life depressive symptoms (LDS) to concurrent episodic memory. (a) The concurrent moderated mediation model. W1, wave 1. The covariates (age, sex, and educational level) were not presented in the figure. The moderated mediation model showed a great fit: χ^2^ = 347.677, df = 57, CFI = 0.937, TLI = 0.917, SRMR = 0.048. *p < 0.05; **p < 0.01; ***p < 0.001. (b) Severe CVDs worsen the negative impacts of LDS on white matter integrity.

Considering the close associations between depressive symptoms, CVDs, WM tract disruption, and GM atrophy related to the thalamus and episodic memory, we estimated a moderated mediation model. As shown in Fig. 3a and online Supplementary Table S7, the CVDs moderated the path between depressive symptoms and WM integrity, but not between depressive symptoms and GM volume. WM integrity mediated the concurrent association between depressive symptoms and episodic memory (*indirect effect* = −0.031, 95% CI −0.070 to −0.008), but this pathway was only significant for those with severe CVDs. Specifically, the interaction between CVDs and depressive symptoms worsened WM integrity (*β* = −0.070, *p* = 0.036). As presented in Fig. 3b, depressive symptoms predicted lower WM integrity only when CVDs were high (one s.d. above mean; *β* = −0.189, *p* < 0.001). To rule out the potential influences of CVDs on cognitive performance, we controlled the effects of CVDs on the whole mediation model, and the moderation effect of CVDs on WM was still significant (online Supplementary Fig. S4).

**Figure 4.**
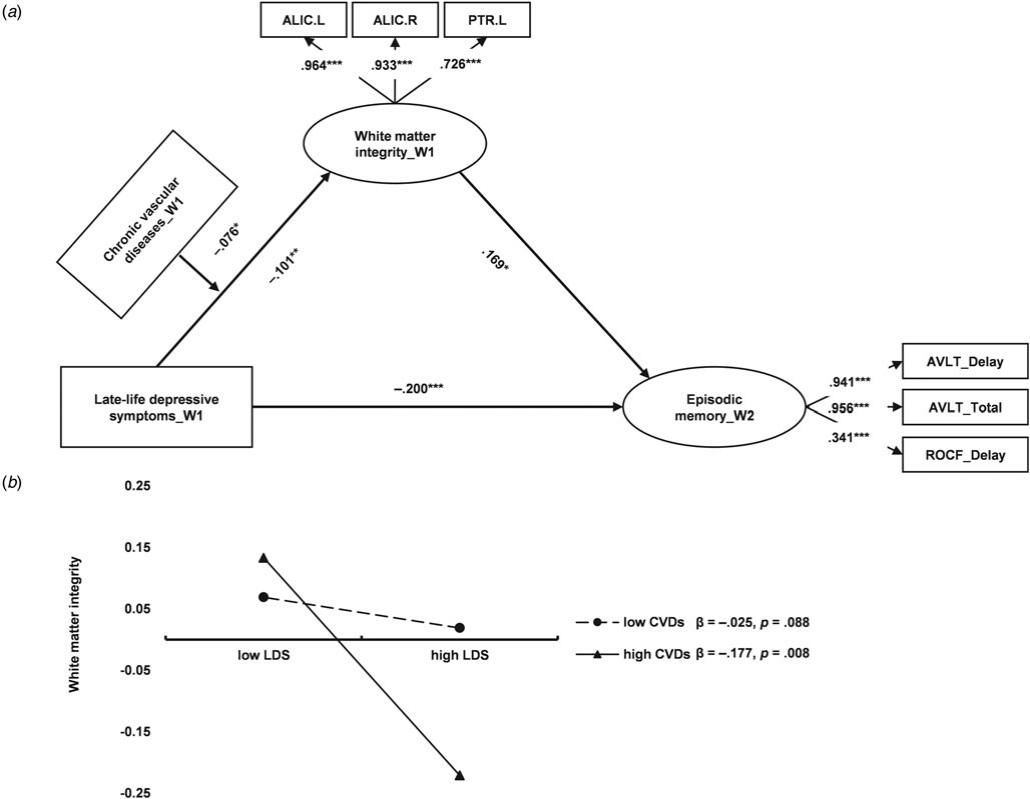
Chronic vascular disease (CVDs) moderates the neural correlates of late-life depressive symptoms (LDS) to subsequent episodic memory. (a) The longitudinal moderated mediation model. W1, wave 1; W2, wave 2. The covariates (age, sex, educational level, collection interval) were not presented in the figure. The moderated mediation model showed a great fit: χ^2^ = 211.438, df = 48, CFI = 0.937, TLI = 0.925, SRMR = 0.075. *p < 0.05; **p < 0.01; ***p < 0.001. (b) Severe CVDs worsen the negative impacts of LDS on white matter integrity.

To further explore the neural correlates linking late-life depressive symptoms and longitudinal cognitive function, we examined a moderated mediation model using longitudinal cognitive data. Our findings showed this model only held when the GM was not included (see Fig. 4a and online Supplementary Table S8). To be specific, WM integrity mediated the longitudinal association between depressive symptoms and episodic memory (*indirect effect* = −0.017, 95% CI −0.045 to −0.002), but this pathway was only significant for those with severe CVDs. Specifically, the interaction between CVDs and depressive symptoms worsened WM integrity (*β* = −0.076, *p* = 0.023). As presented in Fig. 4b, depressive symptoms predicted lower WM integrity only when CVDs were high (one s.d. above mean; *β* = −0.177, *p* = 0.008).

## Discussion

The present study elucidates the neural mechanisms underlying the association between depressive symptoms and both concurrent and longitudinal cognitive performance, as well as explores the moderation of CVDs in linking depressive symptoms to cognitive function. This study found that: (1) depressive symptoms deteriorated cognitive performance in older adults, especially their episodic memory; (2) the thalamus was the region most susceptible to depressive symptoms in this population, and it mediated the episodic memory impairment associated with depressive symptoms; (3) CVDs strengthened the negative association between depressive symptoms and WM integrity, which further correlated with both concurrent and longitudinal cognitive function. These findings suggest that the management of CVDs during aging could be a potential way to mitigate the deleterious effect of depressive symptoms on longitudinal cognitive changes for older adults, and may help prevent the incidence of neurodegenerative diseases such as AD.

Based on a large sample size with comprehensive cognitive assessments, our study observed a strong association between depressive symptoms and episodic memory impairment in old adults, both cross-sectionally and longitudinally. It is reported that the neurobiological substrates play a key role in linking depressive symptoms to memory dysfunction (Butters et al., 2008). Specifically, the depressive symptoms could activate the hypothalamic–pituitary–adrenal (HPA) axis and increase glucocorticoid production, which may further damage the hippocampus and result in memory dysfunction (Sierksma, van den Hove, Steinbusch, & Prickaerts, 2010). In addition, this cortisol-hippocampal pathway has been shown as one of the proposed primary links between depression and dementia (Byers & Yaffe, 2011). Therefore, our findings provided further evidence that older adults with depressive symptoms are at greater risk for Alzheimer's progression. In other words, depressive symptoms may be a prodrome of Alzheimer (Kaup et al., 2016).

Our study found that older adults with depressive symptoms showed widespread WM microstructure damages in projection fibers related to the thalamus, as well as smaller thalamic atrophy with the absence of significant resting-state FC changes. Moreover, we also found the interaction between depressive symptoms and CVDs was only significantly associated with thalamic WM integrity. These findings suggested that depressive symptoms seemed to have a special impact on brain structure, especially on WM. Indeed, structural damages in WM have consistently been reported in older adults with depressive symptoms (Sexton et al., 2012; Shen et al., 2019). In contrast to these findings on WM, the influences of depressive symptoms on GM structure including atrophy and cortical thickness decline were inconsistent (Polyakova et al., 2018; Sexton et al., 2012). Since structural damage to WM is also a major feature of CVD and has a strong correlation with increased depressive symptoms (Haddad et al., 2022), our findings suggest that the comorbidities of psychological and physical disturbances contribute to WM-biased brain change patterns in older adults.

Another interesting finding is that the impacts on the ‘thalamic structural unit’ may underlie the episodic memory deficits associated with depressive symptoms. The thalamus has extensive reciprocal connections to cortical regions (Nakajima & Halassa, 2017) and plays a major role in relaying information. It is reported that regardless of the structural integrity of the cerebral cortex, damage to the thalamus may lead to cognitive impairments by deteriorating information transmission projections (Pardi et al., 2020). Recently, both animal and human studies have confirmed that thalamic-cortical connections are involved in memory encoding and extraction processes, thus influencing memory function (Philp, Korgaonkar, & Grieve, 2014; Wolff, Gibb, Cassel, & Dalrymple-Alford, 2008). Combined with our findings, it is suggested that the depressive symptoms-related episodic memory fluctuations may be the result of deterioration in the integrity of WM involved in memory information transmission. Moreover, a retrospective study found that, compared to the non-depressed group, older adults who later developed depression showed significant memory impairments and WM hyperintensities even before the onset of depression, while hippocampus atrophy was only detected after the onset of depression (Almdahl, Agartz, Hugdahl, & Korsnes, 2022). Our findings are consistent with these results by demonstrating that the episodic memory observed in older adults with depressive symptoms (a subthreshold depression condition) (Biella et al., 2019) was found to be strongly associated with thalamic WM structures but not with the hippocampus.

Also, we found that CVDs were particularly hazardous for individuals exhibiting depressive symptoms, exacerbating the negative effects of depression on the WM microstructure. It has been reported the risk for depression in older adults is more comorbid with CVDs than in young adults (Karel, 1997), suggesting the increasing role of biological vulnerability in amplifying associated adverse consequences during aging (Valkanova & Ebmeier, 2013). At the brain level, previous studies have reported overlaps in the manifestations of depressive symptoms and CVDs in older adults (Direk et al., 2016). These studies collectively indicate the complex interactions between depressive symptoms and CVDs. Thus, the combination of psychological and physical problems may act as a formidable double-whammy, ultimately resulting in severe impairments related to WM.

Additionally, previous research has reported that FC alterations in intra- and inter-networks in DMN, LFP, and RFP were very common in patients with late-life depression (Li et al., 2017). However, the present study did not find a similar pattern of resting-state FC changes in older adults with depressive symptoms. The relation between depressive symptoms and neuroimaging features varied between participants from a patient cohort and a population-based cohort (Binnewies et al., 2022). Usually, older adults with moderate-to-severe depressive symptoms exhibit more extensive and stable brain alterations (Binnewies et al., 2022; Sexton et al., 2012). Since depressive symptoms usually refer to a subclinical condition, the depressive symptoms measured in the current study reflect a relatively lower level of depression compared to clinically diagnosed major depressive disorder (Zhou et al., 2012), which may not be enough to cause significant variations. Additionally, the core manifestation of depression is the impairment of emotional regulation (Loeffler et al., 2019), so in addition to resting-state studies, studies with emotion-specific tasks are encouraged.

In this study, we used data from a large community cohort study to identify the importance of psychological intervention for older adults with depressive symptoms. However, it is crucial to interpret the results with caution due to the relatively high dropout rate and the absence of follow-up neuroimaging assessments. In this study, only 327 participants reported longitudinal cognitive data. To address this concern, we conducted additional analyses comparing demographic variables, cognitive, CVDs, and neurological characteristics between the remaining participants and those who dropped out. Despite many similarities, the remaining participants had a lower incidence of CVDs, potentially influencing the results (online Supplementary Table S9). Future studies should explore the interactions of depressive symptoms and CVDs on cognition using a more robust sample. Also, further investigations with longitudinal MRI data are necessary to validate the neural correlates identified in this study. Moreover, in the present study, even older adults with only mild-to-moderate depressive symptoms demonstrated significant decreases in cognitive performance without simultaneously significant neuroimaging changes. More sensitive experimental designs are needed in the future to reveal the possible compensatory effects, or other pathologies, involved. Lastly, indexes such as WMH and the Framingham score have also traditionally been viewed as markers of CVDs. Future studies could consider including these indexes to explore the role of CVDs severity on the pathway from depressive symptoms to cognitive impairment.

This study highlights the important role of depressive symptoms on cognitive function in older adults. Specifically, a pathological pattern characterized by predominantly WM damage was found in older adults with severe depressive symptoms. Alterations in the thalamus may also underlie the episodic memory deficits resulting from depressive symptoms. Our findings also suggest the comorbidity of psychological disturbances, particularly depressive symptoms, and physical disturbances such as cerebrovascular disease, might aggravate cognitive function in older adults, making them more vulnerable to neurodegenerative diseases such as AD. Thus, it is important to implement community-based interventions to prevent depressive symptoms in older adults, reducing their risk for neurodegenerative diseases. Additionally, when depressive symptoms develop, a potential way to interrupt the linkage between depressive symptoms and episodic memory is through lowering and successful management of CVDs.

In conclusion, our findings provide information not only on the neural correlates of cognitive dysfunction associated with depressive symptoms, but also on the mechanism by which CVDs amplify this negative impact. The findings suggest the necessity of comorbidity management when depressive symptoms develop, as it helps prevent further deterioration of cognitive function. Our findings provide new insights into optimizing the intervention strategies for psychopathological symptoms in the aging population.

## Supporting information

Dai et al. supplementary material Dai et al. supplementary material

## Data Availability

The current study has got permission for data usage from the Beijing Aging Brain Rejuvenation Initiative (BABRI), an ongoing study with an unpublic dataset. The data sharing should be decided upon by the BABRI study group.

## Supplementary material

The supplementary material for this article can be found at https://doi.org/10.1017/S0033291724001612.
