# Supplementary material for: Longitudinal association between depressive symptoms and cognitive function: the neurological mechanism of psychological and physical disturbances on memory
Source: Psychol Med. 2024 Oct 14;54(13):3602–11. doi: 10.1017/S0033291724001612 (PMC11536121; doi:10.1017/S0033291724001612)
Supplement: Dai et al. supplementary material [file S0033291724001612sup001.docx]

Supplementary Materials

**
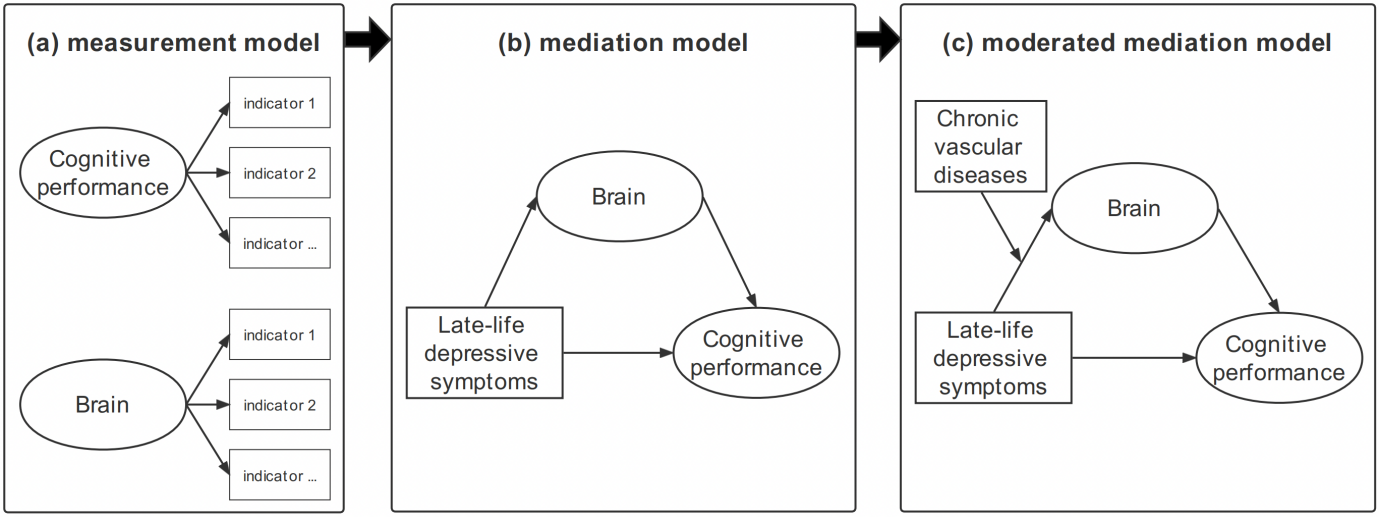
**

**Figure S1** Flow chart of the structural equation modeling.


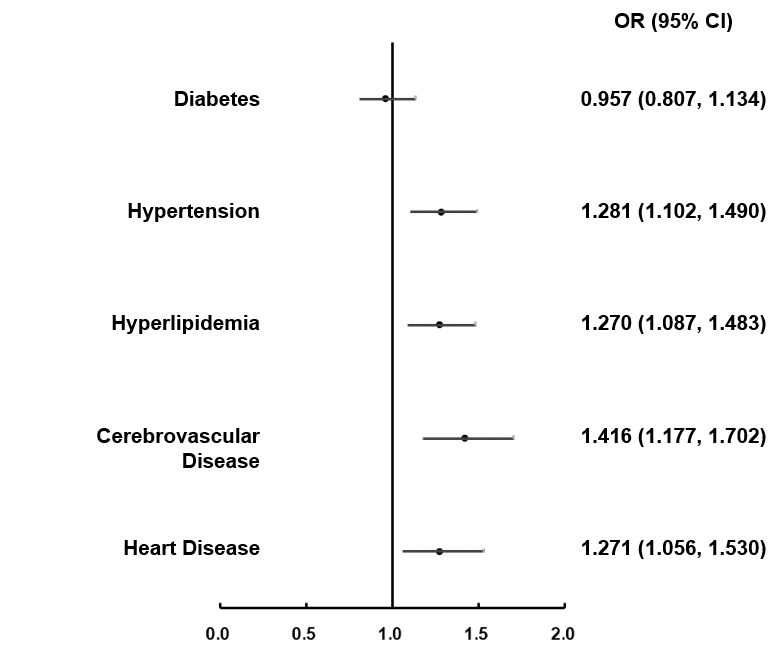


**Figure S2** Forest plot for the vascular risk factor of late-life depressive symptoms.

**
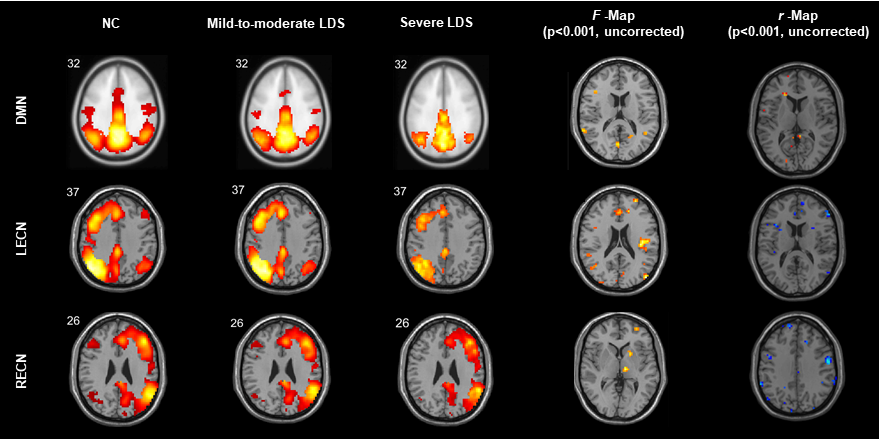
**

**Figure S3** F-maps and r-Maps of the resting-state functional connectivity of networks with late-life depressive symptoms. Adjusted for age, sex, and education. LDS = late-life depressive symptoms; NC, 0 ≤ GDS ≤ 10; Mild-to-moderate LDS, 10 < GDS ≤ 20; Severe LDS, 20 < GDS ≤ 30.


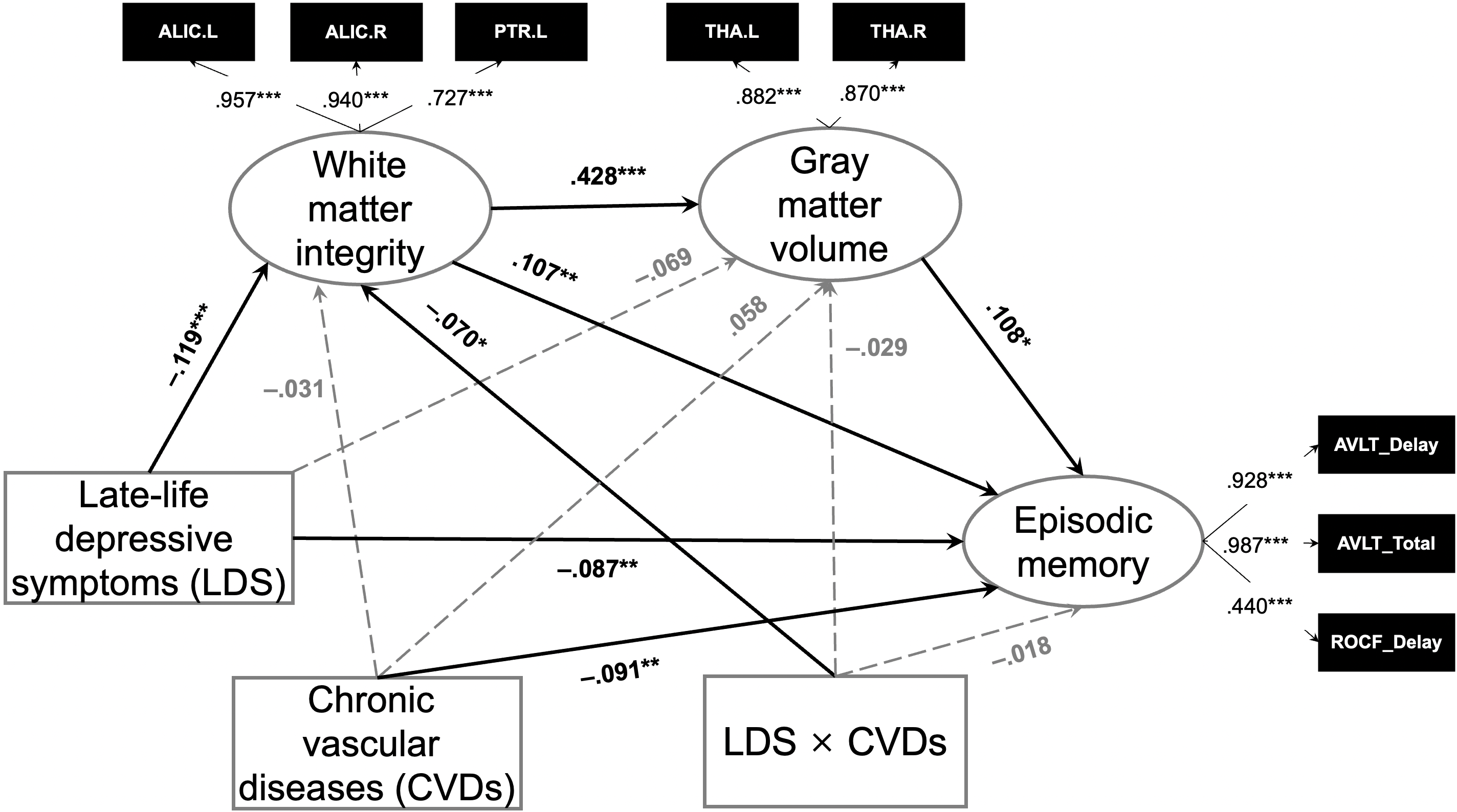


**Fig. S4.** The associations among late-life depressive symptoms, white matter integrity, grey matter volume, chronic vascular diseases, and episodic memory. The covariates (age, sex, and educational level) were not presented in the figure. The moderated mediation model showed a great fit: χ^2^ = 340.147, *df* = 55, CFI = .939, TLI = .915, SRMR = .047.

**p* < .05; ***p* < .01; ****p* < .001.

**Table S1** Study Cohort Characteristics.

|  | ***n*** | **Depressive symptom** | | | **Comorbidities^†^** | | | | |
| --- | --- | --- | --- | --- | --- | --- | --- | --- | --- |
|  |  | **NC** | **Mild-to-moderate**  **LDS** | **Severe**  **LDS** | **Diabetes** | **Hypertension** | **Hyperlipidemia** | **Cerebrovascular**  **Disease** | **Heart**  **Disease** |
| **Age** | | | | | | | | | |
| 50-59 | 961 | 679(70.7%%) | 218(22.7%) | 64(6.7%) | 211(22.4%) | 431(45.9%) | 274(29.1%) | 108(11.5%) | 107(11.4%) |
| 60-69 | 1923 | 1407(73.2%) | 414(21.5%) | 102(5.3%) | 444(23.4%) | 939(49.5%) | 628(33.1%) | 308(16.2%) | 313(16.5%) |
| 70+ | 1225 | 952(77.7%) | 226(18.4%) | 47(3.8%) | 322(26.8%) | 729(60.8%) | 380(31.7%) | 675(16.7%) | 280(23.3%) |
| **Years of education^†^** | | | | | | | | | |
| ≤6 | 354 | 256(72.3%) | 83(23.4%) | 15(4.2%) | 80(23.3%) | 190(55.2%) | 71(20.6%) | 57(16.6%) | 70(20.3%) |
| 6~12 | 2602 | 1848(71.0%) | 589(22.6%) | 165(6.3%) | 652(25.4%) | 1358(53.0%) | 859(33.5%) | 442(17.2%) | 438(17.1%) |
| >12 | 1136 | 922(81.2%) | 182(16.0%) | 32(2.8%) | 237(21.3%) | 543(48.7%) | 349(31.3%) | 171(15.3%) | 188(16.9%) |
| **Sex** | | | | | | | | | |
| Male | 1519 | 1165(76.7%) | 291(19.2%) | 63(4.1%) | 401(26.8%) | 786(52.5%) | 389(26.0%) | 255(17.0%) | 267(17.8%) |
| Female | 2590 | 1873(72.3%) | 567(21.9%) | 150(5.8%) | 576(22.7%) | 1313(51.7%) | 893(35.1%) | 420(16.5%) | 433(17.0%) |

*N* = 4109. Data are *n* (%). LDS = late-life depressive symptoms; NC, 0 ≤ GDS ≤ 10; Mild-to-moderate LDS, 10 < GDS ≤ 20; Severe LDS, 20 < GDS ≤ 30.

**^†^** Missing data: 71 for history of comorbidities and 17 for education.

**Table S2** Difference between MRI sample and longitudinal sample

| **Variables** | **MRI**  **Sample**  **(*n* = 791)** | **Longitudinal**  **Sample**  **(*n* = 327)** | **t/χ^2^** | ***p*** |
| --- | --- | --- | --- | --- |
| **Demographic variables** | | | | |
| Age | 65.427 ± 7.369 | 65.110 ± 6.976 | .665 | .506 |
| Education | 11.366 ± 3.216 | 11.346 ± 3.171 | .097 | .923 |
| Sex (female, %) | 59.7% | 60.6% | .074 | .841 |
| **Diseases (%)** | | | | |
| Diabetes | 24.6% | 21.5% | 1.201 | .313 |
| Hypertension | 48.6% | 43.7% | 2.208 | .146 |
| Hyperlipidemia | 31.8% | 19.7% | 16.583 | < .001 |
| Cerebrovascular disease | 18.5% | 14.2% | 2.990 | .096 |
| Heart disease | 11.3% | 7.4% | 3.826 | .062 |
| number of diseases | 1.347 ±1.237 | 1.065 ± 1.085 | 3.784 | < .001 |
| **Episodic Memory** | | | | |
| AVLT_Delay | 4.592 ± 2.633 | 4.738 ± 2.764 | –.831 | .406 |
| AVLT_Total | 26.057 ± 9.420 | 26.898 ± 9.602 | –1.346 | .178 |
| ROCF_Delay | 12.465 ± 7.077 | 12.884 ± 6.833 | –.897 | .370 |
| **Brain Structural Indicators** | | | | |
| Tha.L | .378 ± .048 | .380 ± .044 | –.714 | .475 |
| Tha.R | .420 ± .053 | .422 ± .048 | –.677 | .499 |
| FA_ALIC.L | .479 ± .036 | .480 ± .030 | –.745 | .456 |
| FA_ALIC.R | .481 ± .035 | .484 ± .028 | –1.782 | .075 |
| FA_PTR.L | .497 ± .040 | .500 ± .036 | –1.063 | .288 |

Note. AVLT = Auditory Verbal Learning Test; ROCF = Rey-Osterrieth Complex Figure Test; Tha = thalamus; L = left; R = right; FA = fractional anisotropy; ALIC = anterior limb of internal capsule; PTR = posterior thalamic radiation, LDS = late-life depressive symptoms.

**Table S3** Z test of the Fisher’s Z transformed correlation coefficients between late-life depressive symptoms and performance of each cognitive domain.

| **Cognitive**  **Domain** | **Processing Speed** | **Executive Function** | **Language** | **Working Memory** | **Spatial Processing** |
| --- | --- | --- | --- | --- | --- |
| **Episodic**  **Memory** | –1.874  (.061) | –2.086  (.037) | –3.739  (< .001) | –4.344  (< .001) | –4.790  (< .001) |
| **Processing**  **Speed** |  | –0.256  (.798) | –1.595  (.111) | –2.569  (.010) | –2.858  (.004) |
| **Executive**  **Function** |  |  | –1.364  (.173) | –2.392  (.017) | –2.561  (.010) |
| **Language** |  |  |  | –1.175  (.240) | –1.393  (.164) |
| **Working**  **Memory** |  |  |  |  | –0.191  (.848) |

**Table S4** White matter integrity differences among the three depressive symptoms groups.

| **Tracts** | **NC** | **Mild-to-moderate**  **LDS** | **Severe**  **LDS** | ***F*** | ***p*** |
| --- | --- | --- | --- | --- | --- |
| **FA** | | | | | |
| MCP | .432 ± .024 | .432 ± .028 | .418 ± .030 | 8.647 | < .001 |
| ACR.L | .375 ± .037 | .377 ± .037 | .356 ± .051 | 9.176 | < .001^†^ |
| ACR.R | .378 ± .037 | .380 ± .038 | .353 ± .055 | 12.628 | < .001^†^ |
| ALIC.L | .479 ± .034 | .482 ± .033 | .457 ± .044 | 11.861 | < .001^†^ |
| ALIC.R | .482 ± .033 | .482 ± .033 | .456 ± .048 | 14.592 | < .001^†^ |
| BCC | .555 ± .042 | .558 ± .043 | .531 ± .053 | 1.835 | < .001^†^ |
| CG.L | .449 ± .039 | .447 ± .039 | .434 ± .040 | 4.094 | 0.017 |
| CG.R | .419 ± .034 | .418 ± .034 | .404 ± .036 | 4.877 | 0.008 |
| CH.L | .339 ± .034 | .343 ± .034 | .336 ± .027 | 0.926 | 0.397 |
| CH.R | .344 ± .032 | .346 ± .032 | .336 ± .030 | 1.916 | 0.148 |
| CP.L | .583 ± .028 | .583 ± .029 | .567 ± .031 | 9.19 | < .001^†^ |
| CP.R | .583 ± .029 | .583 ± .030 | .563 ± .038 | 1.641 | < .001^†^ |
| CST.R | .441 ± .034 | .442 ± .034 | .419 ± .030 | 8.181 | < .001 |
| CST.R | .453 ± .034 | .452 ± .038 | .441 ± .035 | 3.155 | 0.043^†^ |
| EC.L | .353 ± .026 | .356 ± .023 | .342 ± .031 | 5.614 | 0.004 |
| EC.R | .344 ± .024 | .346 ± .022 | .334 ± .030 | 5.332 | 0.005 |
| FX | .294 ± .082 | .303 ± .086 | .288 ± .090 | 1.138 | 0.321 |
| FX/ST | .410 ± .043 | .414 ± .045 | .398 ± .050 | 4.242 | 0.015 |
| FX/ST | .446 ± .040 | .448 ± .040 | .436 ± .042 | 3.886 | 0.021 |
| GCC | .518 ± .035 | .522 ± .038 | .500 ± .042 | 8.296 | < .001^†^ |
| ICP.L | .395 ± .031 | .395 ± .027 | .391 ± .033 | 0.883 | 0.414 |
| ICP.R | .396 ± .032 | .394 ± .029 | .389 ± .033 | 2.362 | 0.095 |
| MCP | .432 ± .024 | .432 ± .028 | .418 ± .030 | 8.647 | < .001^†^ |
| ML.L | .534 ± .034 | .536 ± .030 | .525 ± .032 | 2.896 | 0.056 |
| ML.R | .534 ± .034 | .536 ± .029 | .525 ± .037 | 2.574 | 0.077 |
| PCR.L | .408 ± .033 | .410 ± .033 | .389 ± .041 | 9.459 | < .001^†^ |
| PCR.R | .421 ± .034 | .424 ± .032 | .402 ± .039 | 9.192 | < .001^†^ |
| PCT | .400 ± .036 | .402 ± .037 | .400 ± .038 | 0.044 | 0.957 |
| PLIC.L | .580 ± .031 | .580 ± .031 | .559 ± .038 | 1.02 | < .001^†^ |
| PLIC.R | .574 ± .033 | .575 ± .032 | .556 ± .039 | 7.614 | 0.001 |
| PTR.L | .498 ± .039 | .500 ± .037 | .473 ± .048 | 1.83 | < .001^†^ |
| PTR.R | .507 ± .040 | .510 ± .040 | .488 ± .044 | 6.433 | 0.002 |
| RLIC.L | .526 ± .027 | .527 ± .030 | .509 ± .039 | 7.905 | < .001^†^ |
| RLIC.R | .510 ± .029 | .513 ± .028 | .500 ± .034 | 3.749 | 0.024 |
| SCC | .664 ± .037 | .665 ± .036 | .643 ± .051 | 8.066 | < .001^†^ |
| SCP.L | .500 ± .028 | .499 ± .031 | .482 ± .038 | 8.128 | < .001^†^ |
| SCP.R | .523 ± .032 | .521 ± .033 | .500 ± .042 | 1.617 | < .001^†^ |
| SCR.L | .431 ± .031 | .434 ± .031 | .410 ± .041 | 12.397 | < .001^†^ |
| SCR.R | .425 ± .030 | .428 ± .029 | .406 ± .043 | 11.626 | < .001^†^ |
| SFOF.L | .376 ± .051 | .378 ± .055 | .352 ± .065 | 6.766 | 0.001 |
| SFOF.R | .396 ± .044 | .398 ± .049 | .367 ± .063 | 11.571 | < .001^†^ |
| SLF.L | .419 ± .026 | .420 ± .028 | .396 ± .043 | 13.303 | < .001^†^ |
| SLF.R | .417 ± .027 | .418 ± .027 | .394 ± .043 | 13.078 | < .001^†^ |
| SS.L | .439 ± .031 | .442 ± .032 | .424 ± .040 | 6.391 | 0.002 |
| SS.R | .462 ± .029 | .464 ± .030 | .447 ± .038 | 7.005 | 0.001 |
| TAP.L | .272 ± .048 | .272 ± .052 | .264 ± .052 | 2.261 | 0.105 |
| TAP.R | .354 ± .050 | .358 ± .055 | .340 ± .053 | 3.91 | 0.02 |
| UNC.L | .408 ± .037 | .403 ± .037 | .396 ± .036 | 4.073 | 0.017 |
| UNC.R | .417 ± .034 | .416 ± .035 | .414 ± .034 | 0.397 | 0.673 |
| **MD (10-3)** | | | | | |
| ACR.L | .811 ± .074 | .807 ± .079 | .854 ± .122 | 1.672 | < .001^†^ |
| ACR.R | .819 ± .078 | .814 ± .081 | .876 ± .137 | 14.967 | < .001^†^ |
| ALIC.L | .775 ± .079 | .764 ± .073 | .799 ± .094 | 4.103 | .017 |
| ALIC.R | .784 ± .074 | .774 ± .069 | .820 ± .105 | 7.402 | .001 |
| BCC | .997 ± .074 | .992 ± .081 | 1.037 ± .093 | 11.268 | < .001^†^ |
| CG.L | .774 ± .037 | .771 ± .031 | .783 ± .031 | 3.130 | .044 |
| CG.R | .776 ± .037 | .772 ± .033 | .789 ± .039 | 5.730 | .003 |
| CH.L | .939 ± .115 | .929 ± .113 | .934 ± .092 | .335 | .716 |
| CH.R | .901 ± .090 | .896 ± .102 | .903 ± .084 | 1.003 | .367 |
| CP.L | .793 ± .050 | .789 ± .045 | .792 ± .048 | .169 | .845 |
| CP.R | .824 ± .054 | .820 ± .050 | .831 ± .051 | .614 | .541 |
| CST.R | .599 ± .096 | .599 ± .086 | .623 ± .112 | .901 | .406 |
| CST.R | .594 ± .089 | .595 ± .089 | .602 ± .095 | .104 | .901 |
| EC.L | .832 ± .069 | .820 ± .057 | .846 ± .083 | 3.098 | .046 |
| EC.R | .831 ± .068 | .818 ± .056 | .849 ± .078 | 4.256 | .015 |
| FX | 2.045 ± .389 | 2.012 ± .424 | 2.067 ± .405 | 1.442 | .237 |
| FX/ST | 1.057 ± .168 | 1.052 ± .178 | 1.088 ± .203 | 4.066 | .018 |
| FX/ST | .941 ± .126 | .938 ± .124 | .969 ± .154 | 4.529 | .011 |
| GCC | 1.092 ± .127 | 1.085 ± .140 | 1.113 ± .138 | 2.118 | .121 |
| ICP.L | .233 ± .157 | .238 ± .162 | .236 ± .164 | .028 | .973 |
| ICP.R | .166 ± .176 | .173 ± .183 | .167 ± .176 | .056 | .945 |
| MCP | .333 ± .201 | .339 ± .207 | .346 ± .208 | .066 | .936 |
| ML.L | .415 ± .140 | .418 ± .139 | .417 ± .146 | .036 | .964 |
| ML.R | .385 ± .154 | .389 ± .155 | .393 ± .155 | .017 | .983 |
| PCR.L | .884 ± .096 | .883 ± .111 | .934 ± .137 | 9.327 | < .001^†^ |
| PCR.R | .884 ± .095 | .879 ± .102 | .936 ± .131 | 1.196 | < .001^†^ |
| PCT | .683 ± .084 | .685 ± .083 | .697 ± .068 | .441 | .644 |
| PLIC.L | .723 ± .039 | .721 ± .034 | .744 ± .057 | 7.656 | .001 |
| PLIC.R | .747 ± .036 | .745 ± .039 | .775 ± .073 | 12.706 | < .001^†^ |
| PTR.L | .935 ± .089 | .939 ± .096 | .976 ± .113 | 8.942 | < .001^†^ |
| PTR.R | .889 ± .078 | .884 ± .074 | .913 ± .085 | 4.027 | .018 |
| RLIC.L | .807 ± .063 | .809 ± .065 | .842 ± .098 | 11.038 | < .001^†^ |
| RLIC.R | .831 ± .063 | .827 ± .063 | .858 ± .090 | 7.685 | < .001^†^ |
| SCC | .869 ± .062 | .866 ± .061 | .893 ± .075 | 6.535 | .002 |
| SCP.L | .820 ± .157 | .831 ± .164 | .831 ± .154 | .438 | .645 |
| SCP.R | .734 ± .163 | .748 ± .176 | .747 ± .161 | .575 | .563 |
| SCR.L | .789 ± .074 | .787 ± .075 | .841 ± .127 | 13.657 | < .001^†^ |
| SCR.R | .795 ± .066 | .793 ± .076 | .856 ± .150 | 17.415 | < .001^†^ |
| SFOF.L | .814 ± .160 | .809 ± .154 | .877 ± .199 | 5.610 | .004 |
| SFOF.R | .798 ± .111 | .793 ± .151 | .882 ± .241 | 11.229 | < .001^†^ |
| SLF.L | .759 ± .048 | .756 ± .051 | .793 ± .092 | 1.928 | < .001^†^ |
| SLF.R | .779 ± .050 | .777 ± .051 | .819 ± .096 | 13.965 | < .001^†^ |
| SS.L | .939 ± .096 | .937 ± .100 | .983 ± .112 | 1.271 | < .001^†^ |
| SS.R | .907 ± .069 | .908 ± .076 | .948 ± .082 | 14.448 | < .001^†^ |
| TAP.L | 2.057 ± .206 | 2.045 ± .235 | 2.090 ± .218 | 1.752 | .174 |
| TAP.R | 1.709 ± .197 | 1.708 ± .218 | 1.756 ± .206 | 3.933 | .020 |
| UNC.L | .767 ± .056 | .774 ± .052 | .776 ± .044 | 2.830 | .060 |
| UNC.R | .796 ± .055 | .792 ± .049 | .800 ± .043 | .686 | .504 |

Notes: Adjusted for age, sex and years of education. LDS, late-life depressive symptoms; NC, 0 ≤ GDS ≤ 10; Mild-to-moderate LDS, 10 < GDS ≤ 20; Severe LDS, 20 < GDS ≤ 30; FA, fractional anisotropy; MD, mean diffusivity. ^†^*Post hoc* paired comparisons showed significant group differences between severe LDS and NC as well as between severe LDS and Mild-to-moderate LDS. Abbreviations for white matter tracts are shown in Table S10.

**Table S5** The correlations between late-life depressive symptoms and FA of the atlas-based tract ROIs.

| **Tracks** | ***M* ± *SD*** | ***r*** | ***p*^*^** |
| --- | --- | --- | --- |
| ACR.L | .375 ± .038 | –.139 | .0001 |
| ACR.R | .377 ± .039 | –.160 | < .0001 |
| ALIC.L | .479 ± .034 | –.130 | .0004 |
| ALIC.R | .480 ± .034 | –.156 | < .0001 |
| BCC | .555 ± .043 | –.131 | .0004 |
| CP.L | .583 ± .029 | –.129 | .0004 |
| MCP | .431 ± .026 | –.136 | .0002 |
| PCR.L | .408 ± .033 | –.136 | .0002 |
| PTR.L | .497 ± .040 | –.151 | < .0001 |
| SCC | .663 ± .038 | –.126 | .0006 |
| SCP.R | .521 ± .033 | –.141 | .0001 |
| SFOF.L | .375 ± .053 | –.123 | .0008 |
| SFOF.R | .395 ± .047 | –.157 | < .0001 |
| SLF.L | .418 ± .028 | –.122 | .0009 |
| SLF.R | .416 ± .029 | –.126 | .0006 |

Notes: Adjusted for age, sex and years of education. FA, fraction anisotropy. *M* = Mean; *SD* = Standard deviation. ^*^Bonferroni corrected (*p* < .001, 0.05/48). Abbreviations for white matter tracts are shown in Table S10.

**Table S6** Thalamic volume differences among the three depressive symptoms groups

|  | **NC** | **Mild-to-moderate**  **LDS** | **Severe**  **LDS** | ***F*** | ***p*** |
| --- | --- | --- | --- | --- | --- |
| Thalamus.L | .379 ± .047 | .376 ± .045 | .361 ± .072 | 5.052^*^ | .007^†^ |
| Thalamus.R | .422 ± .050 | .418 ± .051 | .398 ± .085 | 4.626^*^ | .010^†^ |

Notes: Adjusted for age, sex and years of education. LDS = late-life depressive symptoms; NC, 0 ≤ GDS ≤ 10; Mild-to-moderate LDS, 10 < GDS ≤ 20; Severe LDS, 20 < GDS ≤ 30; L = left; R = right. ^*^ *p* < .005. ^†^*Post hoc* paired comparisons showed the volume of bilateral thalamus of paricipants with severe LDS was significantly lower than those with mild-to-moderate LDS or in the NC group.

**Table S7** A moderated mediation model among LDS, CVDs, brain alterations, and concurrent EM

|  | **white matter**  **integrity** | | | **grey matter**  **volume** | | | **concurrent**  **EM** | | |
| --- | --- | --- | --- | --- | --- | --- | --- | --- | --- |
| **predictors** | **β** | ***SE*** | ***p*** | **β** | ***SE*** | ***p*** | **β** | ***SE*** | ***p*** |
| Age | –.365 | .041 | < .001 | –.096 | .047 | .040 | –.258 | .039 | < .001 |
| Sex | .027 | .039 | .487 | .103 | .055 | .060 | .091 | .035 | .009 |
| Education | –.023 | .039 | .561 | .121 | .036 | .001 | .191 | .033 | < .001 |
| LDS | –.119 | .037 | .001 | –.069 | .041 | .090 | –.076 | .032 | .019 |
| white matter integrity |  |  |  | .428 | .058 | < .001 | .101 | .039 | .010 |
| grey matter volume |  |  |  |  |  |  | .118 | .044 | .007 |
| CVDs | –.031 | .034 | .365 | .060 | .036 | .093 |  |  |  |
| LDS × CVDs | –.070 | .033 | .036 | –.029 | .041 | .483 |  |  |  |
| *R*^2^ | .146 | .028 | < .001 | .257 | .041 | < .001 | .183 | .026 | < .001 |

Note. LDS = late-life depressive symptoms; CVDs = chronic vascular diseases; EM = concurrent episodic memory.

**Table S8** A moderated mediation model among LDS, CVDs, brain alterations, and subsequent EM

|  | **white matter integrity** | | | **subsequent EM** | | |
| --- | --- | --- | --- | --- | --- | --- |
| **predictors** | **β** | ***SE*** | ***p*** | **β** | ***SE*** | ***p*** |
| Age | –.367 | .042 | < .001 | –.232 | .067 | .001 |
| Sex | .006 | .040 | .874 | .157 | .052 | .003 |
| Education | –.039 | .040 | .333 | .162 | .061 | .008 |
| Collection interval | .222 | .070 | .002 | –.024 | .067 | .714 |
| LDS | –.101 | .038 | .007 | –.200 | .052 | < .001 |
| White matter integrity |  |  |  | .169 | .080 | .035 |
| CVDs | –.044 | .034 | .202 |  |  |  |
| LDS × CVDs | –.076 | .033 | .023 |  |  |  |
| *R*^2^ | .193 | .037 | < .001 | .190 | .046 | < .001 |

Note. LDS = late-life depressive symptoms; WM = white matter integrity; CVDs = chronic vascular diseases; EM = episodic memory.

**Table S9** Difference between retaining and dropping participants

| **Variables** | **Retaining participants**  **(*n* = 327)** | **Dropping participants**  **(*n* = 464)** | **t/χ^2^** | ***p*** |
| --- | --- | --- | --- | --- |
| **Demographic variables** | | | | |
| Age | 65.110 ± 6.976 | 65.651 ± 7.633 | –1.016 | .310 |
| Education | 11.346 ± 3.171 | 11.380 ± 3.250 | –.151 | .880 |
| Sex (female, %) | 60.6% | 59.1% | –.179 | .713 |
| **Diseases (%)** | | | | |
| Diabetes | 21.5% | 26.8% | 2.843 | .109 |
| Hypertension | 43.7% | 52.1% | 5.349 | .024 |
| Hyperlipidemia | 19.7% | 40.4% | 37.631 | < .001 |
| Cerebrovascular disease | 14.2% | 21.5% | 6.868 | .009 |
| Heart disease | 7.4% | 14.1% | 8.455 | .004 |
| number of diseases | 1.065 ± 1.085 | 1.549 ± 1.299 | 5.661 | < .001 |
| **Episodic Memory** | | | | |
| AVLT_Delay | 4.738 ± 2.764 | 4.489 ± 2.535 | 1.308 | .191 |
| AVLT_Total | 26.898 ± 9.602 | 25.464 ± 9.255 | 2.107 | .035 |
| ROCF_Delay | 12.884 ± 6.833 | 12.170 ± 7.236 | 1.378 | .169 |
| **Brain Structural Indicators** | | | | |
| Tha.L | .380 ± .044 | .376 ± .052 | 1.116 | .265 |
| Tha.R | .422 ± .048 | .418 ± .056 | 1.058 | .291 |
| FA_ALIC.L | .480 ± .030 | .478 ± .039 | 1.149 | .251 |
| FA_ALIC.R | .484 ± .028 | .478 ± .039 | 2.521 | .012 |
| FA_PTR.L | .500 ± .036 | .495 ± .043 | 1.636 | .102 |

Note. AVLT = Auditory Verbal Learning Test; ROCF = Rey-Osterrieth Complex Figure Test; Tha = thalamus; L = left; R = right; FA = fractional anisotropy; ALIC = anterior limb of internal capsule; PTR = posterior thalamic radiation, LDS = late-life depressive symptoms.

**Table S10.** Abbrevations of white matter tracts

| **Index** | **Tracs** | **Abbr.** |
| --- | --- | --- |
| 1 | Anterior_corona_radiata | ACR.L |
| 2 | Anterior_corona_radiata_R | ACR.R |
| 3 | Anterior_limb_of_internal_capsule_L | ALIC.L |
| 4 | Anterior_limb_of_internal_capsule_R | ALIC.R |
| 5 | Body_of_corpus_callosum | BCC |
| 6 | Cerebral_peduncle_L | CP.L |
| 7 | Cerebral_peduncle_R | CP.R |
| 8 | Cingulum_(cingulate_gyrus)_L | CG.L |
| 9 | Cingulum_(cingulate_gyrus)_R | CG.R |
| 10 | Cingulum_(hippocampus)_L | CH.L |
| 11 | Cingulum_(hippocampus)_R | CH.R |
| 12 | Corticospinal_tract_L | CST.R |
| 13 | Corticospinal_tract_R | CST.R |
| 14 | External_capsule_L | EC.L |
| 15 | External_capsule_R | EC.R |
| 16 | Fornix_(column_and_body_of_fornix) | FX |
| 17 | Fornix_(cres)_/_Stria_terminalis_(can_not_be_resolved_with_current_resolution)_L | F/ST.L |
| 18 | Fornix_(cres)_/_Stria_terminalis_(can_not_be_resolved_with_current_resolution)_R | F/ST.R |
| 19 | Genu_of_corpus_callosum | GCC |
| 20 | Inferior_cerebellar_peduncle_L | ICP.L |
| 21 | Inferior_cerebellar_peduncle_R | ICP.R |
| 22 | Medial_lemniscus_L | ML.L |
| 23 | Medial_lemniscus_R | ML.R |
| 24 | Middle_cerebellar_peduncle | MCP |
| 25 | Pontine_crossing_tract_(a_part_of_MCP) | PCT |
| 26 | Posterior_corona_radiata_L | PCR.L |
| 27 | Posterior_corona_radiata_R | PCR.R |
| 28 | Posterior_limb_of_internal_capsule_L | PLIC.L |
| 29 | Posterior_limb_of_internal_capsule_R | PLIC.R |
| 30 | Posterior_thalamic_radiation_(include_optic_radiation)_L | PTR.L |
| 31 | Posterior_thalamic_radiation_(include_optic_radiation)_R | PTR.R |
| 32 | Retrolenticular_part_of_internal_capsule_L | RLIC.L |
| 33 | Retrolenticular_part_of_internal_capsule_R | RLIC.R |
| 34 | Sagittal_stratum_(include_inferior_longitidinal_fasciculus_and_inferior_fronto-occipital_fasciculus)_L | SS.L |
| 35 | Sagittal_stratum_(include_inferior_longitidinal_fasciculus_and_inferior_fronto-occipital_fasciculus)_R | SS.R |
| 36 | Splenium_of_corpus_callosum | SCC |
| 37 | Superior_cerebellar_peduncle_L | SCP.L |
| 38 | Superior_cerebellar_peduncle_R | SCP.R |
| 39 | Superior_corona_radiata_L | SCR.L |
| 40 | Superior_corona_radiata_R | SCR.R |
| 41 | Superior_fronto-occipital_fasciculus_(could_be_a_part_of_anterior_internal_capsule)_L | SFOF.L |
| 42 | Superior_fronto-occipital_fasciculus_(could_be_a_part_of_anterior_internal_capsule)_R | SFOF.R |
| 43 | Superior_longitudinal_fasciculus_L | SLF.L |
| 44 | Superior_longitudinal_fasciculus_R | SLF.R |
| 45 | Tapetum_L | TAP.L |
| 46 | Tapetum_R | TAP.R |
| 47 | Uncinate_fasciculus_L | UNC.L |
| 48 | Uncinate_fasciculus_R | UNC.R |
